# Bacterial and Fungal Infections in Persons Who Inject Drugs — Western New York, 2017

**DOI:** 10.15585/mmwr.mm6826a2

**Published:** 2019-07-05

**Authors:** Kathleen P. Hartnett, Kelly A. Jackson, Christina Felsen, Robert McDonald, Ana Cecilia Bardossy, Runa H. Gokhale, Ian Kracalik, Todd Lucas, Olivia McGovern, Chris A. Van Beneden, Michael Mendoza, Michele Bohm, John T. Brooks, Alice K. Asher, Shelley S. Magill, Anthony Fiore, Debra Blog, Elizabeth M. Dufort, Isaac See, Ghinwa Dumyati

**Affiliations:** ^1^Epidemic Intelligence Service, CDC; ^2^Division of Healthcare Quality Promotion, National Center for Emerging and Zoonotic Infectious Diseases, CDC; ^3^Emerging Infections Program, University of Rochester Medical Center, Rochester, New York; ^4^New York State Department of Health; ^5^Division of Bacterial Diseases, National Center for Immunization and Respiratory Diseases, CDC; ^6^Monroe County Department of Public Health, New York; ^7^Division of Unintentional Injury Prevention, National Center for Injury Prevention and Control, CDC; ^8^Division of HIV/AIDS Prevention, National Center for HIV/AIDS, Viral Hepatitis, STD, and TB Prevention, CDC; ^9^Office of Policy, Planning and Partnerships, Office of the Director, National Center for HIV/AIDS, Viral Hepatitis, STD, and TB Prevention, CDC.

During 2014–2017, CDC Emerging Infections Program surveillance data reported that the occurrence of invasive methicillin-resistant *Staphylococcus aureus* (MRSA) infections associated with injection drug use doubled among persons aged 18–49 years residing in Monroe County in western New York.* Unpublished surveillance data also indicate that an increasing proportion of all *Candida* spp. bloodstream infections in Monroe County and invasive group A *Streptococcus* (GAS) infections in 15 New York counties are also occurring among persons who inject drugs. In addition, across six surveillance sites nationwide, the proportion of invasive MRSA infections that occurred in persons who inject drugs increased from 4.1% of invasive MRSA cases in 2011 to 9.2% in 2016 (*1*). To better understand the types and frequency of these infections and identify prevention opportunities, CDC and public health partners conducted a rapid assessment of bacterial and fungal infections among persons who inject drugs in western New York. The goals were to assess which bacterial and fungal pathogens most often cause infections in persons who inject drugs, what proportion of persons who inject use opioids, and of these, how many were offered medication-assisted treatment for opioid use disorder. Medication-assisted treatment, which includes use of medications such as buprenorphine, methadone, and naltrexone, reduces cravings and has been reported to lower the risk for overdose death and all-cause mortality in persons who use opioids (*2*,*3*). In this assessment, nearly all persons with infections who injected drugs used opioids (97%), but half of inpatients (22 of 44) and 12 of 13 patients seen only in the emergency department (ED) were not offered medication-assisted treatment. The most commonly identified pathogen was *S. aureus* (80%), which is frequently found on skin. Health care visits for bacterial and fungal infections associated with injection opioid use are an opportunity to treat the underlying opioid use disorder with medication-assisted treatment. Routine care for patients who continue to inject should include advice on hand hygiene and not injecting into skin that has not been cleaned or to use any equipment contaminated by reuse, saliva, soil, or water (*4*,*5*). 

The team obtained and reviewed records for hospital admissions and ED visits during April 1–June 30, 2017, from a convenience sample of five hospitals in western New York. Patients of any age who had 1) positive cultures for *S. aureus* (excluding nasal specimens), *Candida* spp. in blood, or GAS from a normally sterile site or 2) diagnostic codes related to substance use and a bacterial or fungal pathogen or infection^†^ were included. Injection drug use was defined as patient self-report of injection drug use; health care worker, relative, or friend report that the person injected drugs; or observation of injection equipment in the patient’s room or belongings or skin lesions indicative of injection drug use (track marks). Demographic information, infection sites, bacterial and fungal pathogens, history of human immunodeficiency virus (HIV), hepatitis B and C, and clinical outcomes were abstracted from medical records for all patients with injection drug use. Information on substance use history and treatment was collected for a subset of persons whose infections were identified from *S. aureus*, *Candida* spp., or GAS culture. A chi-squared test was performed using SAS (version 9.4; SAS Institute) to compare the proportion of patients seen only in the ED to the proportion of hospitalized patients who were offered medication-assisted treatment. To assess the sensitivity of identifying patients with infections using diagnostic codes alone, the proportion of patients who injected drugs identified by positive cultures who also had diagnostic codes for both substance use and a bacterial or fungal pathogen or infection was calculated. 

Among 1,002 patients who met either inclusion criterion, medical records for 111 (11%) documented injection drug use during the previous 12 months. The median age of these persons was 32 years (range = 18–68 years); 61% were women (Table). Skin and soft tissue infections accounted for 82 (74%) infections, and endocarditis accounted for 16 (14%). Among skin and soft tissue infections, 50 (61%) were documented to be at an injection site, and 12 (15%) were not at an injection site. For 20 patients (24%), the medical record did not document whether the infection was at a site where the person injected drugs. Overall, 79 persons (71%) were hospitalized, of whom 19 (24%) were hospitalized for ≥30 days. Four (4%) patients died before leaving the hospital. Thirty-three (30%) patients left the hospital against medical advice, including 13 (41%) of 32 persons seen only in the ED and 20 (25%) of 79 persons admitted to the hospital.

**TABLE:** Characteristics of persons who inject drugs and were evaluated in emergency departments or admitted to the hospital for bacterial or fungal infections (N = 111) — western New York, 2017*

Characteristic	No. (%)
**Female sex**	68 (61)
**Median age (range), yrs**	32 (18–68)
**Microbiology**	
Not cultured	15 (14)
No relevant cultures positive	26 (23)
Organism identified	70 (63)
**Organism**^†^ **(n = 70)**	
*Staphylococcus aureus^§^*	56 (80)
*Streptococcus* spp.^¶^	11 (16)
Other bacteria**	22 (31)
*Candida* spp.	4 (6)
Fungal, not otherwise specified	1 (1)
**Infection type** ^††^	
Skin and soft tissue**^§§^**	82 (74)
Endocarditis	16 (14)
Osteomyelitis	6 (5)
Pneumonia	5 (5)
Bacteremia without other infection type	3 (3)
Empyema	3 (3)
Septic arthritis	2 (2)
Other	4 (4)
**Treatment outcome**	
Died during hospital visit	4 (4)
Admitted to the hospital	79 (71)
Left against medical advice	33 (30)
Inpatients (% of 79 admissions)	20 (25)
ED only visits (% of 32 ED-only visits)	13 (41)
**Length of stay**	
All admitted patients: median (Q1–Q3) days	7 (4–29)
Admitted patients who did not leave against medical advice: median (Q1–Q3) days	9 (4–36)
All admitted patients: hospitalized >30 days	19 (24)
**Drug used (n = 59)** ^¶¶^	
Opioids and cocaine	41 (69)
Opioids only	13 (22)
Opioids, cocaine, and methamphetamine	2 (3)
Opioids and methamphetamine	1 (2)
Cocaine only	1 (2)
Cocaine and methamphetamine	1 (2)
**Offered medication-assisted treatment for opioid use disorder during visit (n = 57 using opioids)**	23 (40)
Admitted patients (% of 44 admissions of persons with opioid use)	22 (50)
ED-only visits (% of 13 ED-only visits of persons with opioid use)	1 (8)
Bloodborne pathogens***	
Human immunodeficiency virus	7 (6)
Hepatitis B virus	4 (4)
Hepatitis C virus	41 (37)
**Patients with diagnostic codes for both infection syndrome and substance use (n = 53 identified by culture)** ^†††^	39 (74)

**Abbreviations**: ED = emergency department; Q1 = quartile 1; Q3 = quartile 3.

* April 1–June 30, 2017, at five hospitals in western New York for patients of any age who injected drugs and had 1) positive cultures for *S*. *aureus* (excluding nasal specimens), *Candida* spp. in blood, or group A *Streptococcus* from a normally sterile site or 2) diagnostic codes including both substance use disorder and a bacterial or fungal pathogen or infection.

^†^ Percentages are calculated among 70 patients with an organism identified and do not sum to 100 because 13 of 70 persons (19%) had an infection with more than one organism identified.

^§^ 30 methicillin-resistant *S. aureus* and 26 methicillin-sensitive *S. aureus*.

^¶^ Eight viridans group *Streptococcus*, two group A *Streptococcus*, and one group C *Streptococcus*.

** 12 gram-negative bacteria including *Enterobacter cloacae* (two), *Eikenella corrodens*, *Escherichia coli*, *Leclercia* spp., *Moraxella catarrhalis*, *Serratia marcescens*, *Sphingomonas paucimobilis*, unspecified gram-negative rods (three), unspecified anaerobic gram-negative cocci; 10 gram-positive bacteria including *Actinomyces* spp. (two), coagulase negative *Staphylococcus* (two [possible contaminants]), *Aerococcus viridans*, *Bacillus* spp., *Corynebacterium* spp., *Granulicatella* spp., unspecified gram-positive cocci chain, and unspecified gram-positive bacilli.

^††^ Infection types are not mutually exclusive, with the exception of bacteremia without other infection type, and other, which includes only patients without another infection type. Other includes intra-abdominal abscess, supraclavicular lymphadenitis, spontaneous bacterial peritonitis, and subacute fungal cerebritis with meningoencephalitis.

^§§^ Includes necrotizing fasciitis (two). Among 82 skin and soft tissue infections, 50 (61%) were documented in the medical record to be at a known injection site, 12 (15%) were not at an injection site, and for 20 (24%), it was unknown or not documented whether the infection was at an injection site.

^¶¶^ Patients for whom drug use data were collected.

*** Chronic infection with hepatitis B virus (HBV), hepatitis C virus (HCV), or human immunodeficiency virus (HIV) noted in the medical history, or the patient had 1) positive HBV surface antigen, 2) positive HCV antibody without RNA tested (could indicate resolved or cured infection) or detectable HCV viral load, or 3) positive HIV test in the record.

^†††^ Excludes six patients without diagnostic codes available for review in the medical record.

Of 70 patients with at least one pathogen identified from a clinical culture, 13 (19%) had a polymicrobial infection. The most common bacterial and fungal pathogens were *S. aureus* (56; 80%); streptococci (11; 16%), including eight viridans group and two GAS; and *Candida* spp. (4; 6%). The most common bloodborne pathogen identified^§^was hepatitis C virus; 41 (37%) patients had a current or previous hepatitis C virus infection documented in the medical record; seven (6%) had a history of HIV infection, and four (4%) had hepatitis B virus infection.

Among a subset of 59 (53%) patients with *S. aureus*, *Candida* spp., or GAS infections from whom drug use data were collected, 57 (97%) used opioids, including 50 who injected opioids and seven with an unknown route of opioid administration. Among 44 inpatients, 22 (50%) were offered medication-assisted treatment for opioid use disorder, whereas one of 13 (8%) persons seen only in the ED was offered medication-assisted treatment (p-value = 0.01). Most patients with an infection identified by culture (74%) also had diagnostic codes for both substance use and an infection or pathogen. 

## Discussion

On average, at least one person with a bacterial or fungal infection who also injects drugs visited one of the five assessed hospitals every day during the analysis period. This investigation highlights the importance of preventing opioid misuse, treating opioid use disorder, and emphasizing the risks of bacterial and fungal infections as well as bloodborne pathogens during care of persons who inject drugs. In this assessment, infections related to injection drug use most often occurred at the site of injection and were predominantly caused by common skin and mouth flora that are introduced during injection. Infections related to injection also included invasive infections, such as endocarditis. Many of the infections required prolonged hospital stays, with 24% of patients hospitalized for at least 30 days. Although nearly all patients injected opioids, many were not offered medication-assisted treatment for opioid use disorder. Those seen only in the ED were less likely to be offered medication-assisted treatment than inpatients.

This assessment was limited to western New York; however, bacterial and fungal infections might also occur frequently in other communities in the United States. Although the prevalence of injection drug use is unknown, the age-adjusted rate of overdose deaths involving any drug in Monroe County, New York, where four of the five hospitals were located, was 24.5 per 100,000 residents in 2016,^¶^ compared with 19.8 drug overdose deaths per 100,000 residents for the United States as a whole (*6*).

The findings in this report are subject to at least three limitations. First, the number of bacterial and fungal infections among persons who inject drugs was likely underestimated because the data did not include outpatient visits or infections in persons who did not seek health care. Second, medical records do not always specify the route of drug administration; records indicating that the patient used drugs but did not document injection were excluded, which also might underestimate the number of persons injecting. Finally, the method of identifying infections could bias the distribution of pathogens or infection types. *S. aureus*, *Candida* spp. and GAS infections were identified by both culture and diagnostic codes. Infections with other pathogens or without a pathogen identified were identified by diagnostic codes only, and therefore were more likely to be missed. However, evidence suggests that most infections were identified through diagnostic codes. Among *S. aureus*, *Candida* spp., and GAS infections identified by culture, 74% had codes for both an infection syndrome and substance use.

Routine care for patients who continue to inject should include advice on hand hygiene and not injecting into skin that has not been cleaned or to use any equipment contaminated by reuse, saliva, soil, or water (*4*,*5*). Risk factors for bacterial and fungal infections found in other recent assessments include skin breakdown and limited access to clean running water and showers (*7*). Where legal, syringe service programs can provide referrals to treatment for substance use disorder, clean equipment, and education about safer injection practices. Other services, such as prompt wound care, laundry, and showers could also help prevent serious bacterial and fungal infections (*8*). Because some persons who misuse prescription opioids transition to injecting opioids, primary prevention strategies that can reduce the risk for opioid misuse and potential subsequent infection from unsafe injection practices include appropriate opioid prescribing practices and efforts to ensure access to nonopioid treatments for pain (*9*). Medication-assisted treatment addresses the underlying opioid use disorder through decreased cravings and prevents infections by reducing injection drug use. Initiating medication-assisted treatment when persons who inject opioids are found to have a bacterial or fungal infection might also improve retention of these patients in treatment for both the infection and substance abuse (*10*). Hospitalizations and ED visits for these infections are opportunities to link patients to treatment for opioid use disorder and prevent recurrent infections.

**Summary**

**What is already known about this topic?**

Bacterial and fungal infections among persons who inject drugs are increasing.

**What is added by this report?**

Among a sample of persons in western New York who inject drugs and were hospitalized or treated in the emergency department for a bacterial and fungal infection, *Staphylococcus aureus* was the most common pathogen. Nearly all persons with such infections injected opioids; most were not offered medication-assisted treatment to reduce injection drug use.

**What are the implications for public health practice?**

Health care visits for bacterial and fungal infections represent an opportunity to treat the underlying opioid use disorder with medication-assisted treatment. Because many infections are caused by skin flora such as *S. aureus*, injecting without first cleaning the injection site and washing hands increases the risk for bacterial and fungal infections.

## Footnotes

* https://idsa.confex.com/idsa/2018/webprogram/Paper72151.html.

† Infection and substance use related codes: A18.84, A31*, A32.82, A39*, A39.51, A41*–A44*, A46*, A48*, A49*, A54.83, B37* –B46*, B49, B95*–B96*, B99.8*, B99.9*, D73.3, E06.0, E32.1, G06*, H05*, I08, I33*, I38, I39, I40.0, I51.89, 172.9, I76, I80*, I96, J85*, J86*, K11.3, K12.2, K13.0, K61*, K65*, K68.1*, L01*–L04*, L08*, L97*, L98.4*, M00*, M01*, M27.2, M46.2*, M46.3*–M46.5, M65.0*, M71.0*, M72.6, M72.8, M86*, N15.1, R65.2*, R78.81, T79.8XXA, T80.2*, Z16, Z79.2. ICD-10 substance use related codes: F11*, F13*–F16*, F18*, F19*, T40*.

§ Hepatitis B virus (HBV), hepatitis C virus (HCV), HIV chronic or acute infection noted in the medical history, or the patient had 1) positive HBV surface antigen, 2) positive HCV antibody without RNA tested (could indicate resolved or cured infection) or detectable HCV viral load, or 3) positive HIV test in the record.

¶ New York State Opioid Dashboard. https://www.health.ny.gov/statistics/opioid.
